# The Role of Local Therapy for Oligo-Progressive Disease in Oncogene-Addicted Non-Small-Cell Lung Cancer

**DOI:** 10.1016/j.adro.2024.101516

**Published:** 2024-04-14

**Authors:** David Chun Cheong Tsui, Douglas E. Holt, Tejas Patil, Alyse Staley, Dexiang Gao, Brian D. Kavanagh, Erin L. Schenk, Chad G. Rusthoven, D. Ross Camidge

**Affiliations:** a Division of Medical Oncology, Department of Medicine, University of Colorado Cancer Center, Denver, Colorado; b Department of Radiation Oncology, University of Colorado Anschutz Medical Campus, Denver, Colorado; c Department of Pediatrics, University of Colorado School of Medicine; Biostatistics and Bioinformatics Shared Resource, University of Colorado Cancer Center, Aurora, Colorado

## Abstract

**Purpose:**

We first described the role of local radiation therapy (LT) for oligoprogressive disease (OPD) on targeted therapy in 2012. Here, we present an updated and larger data set and extend the analysis beyond EGFR and ALK.

**Methods:**

A retrospective review of patients with metastatic NSCLC harboring *EGFR/BRAF* V600E mutations, or *ALK/ROS1/RET* rearrangements, who had OPD on respective tyrosine-kinase inhibitor (TKI) and treated with LT was performed. OPD was defined as disease progression on therapy in ≤5 sites. PFS1 (progression-free survival 1) was defined as time from initiation of TKI-containing regimen to the first course of LT for OPD. Subsequent PFS times (eg, PFS2, PFS3) were defined as time from prior LT to subsequent LT, switch of systemic therapy, death, or loss to follow-up, whichever occurred first. Extended-PFS was defined as time from the first day of the first LT course to the day of change in systemic therapy, death, or loss to follow-up, whichever came first.

**Results:**

Eighty-nine patients were identified. In 75.4% of the LT courses, a single lesion was treated. Median PFS1 was 10.2 months (95% CI, 8.7-13.1) and median Extended-PFS was 6.7 months (95% CI, 4.9-8.3). Extended-PFS was similar across different oncogenic drivers; 51.4% of patients who underwent LT to a single site had only 1 site on next disease progression.

**Conclusions:**

LT is effective in prolonging treatment duration on TKI in oncogene-addicted NSCLC across multiple oncogenes.

## Introduction

Oligo-progressive disease (OPD), a state where disease progression on treatment occurs at only a few (usually 5 or fewer) central nervous system (CNS) or extra-CNS sites for patients with otherwise drug-controlled metastatic disease, is common in oncogene-addicted non-small-cell lung cancer (NSCLC). OPD is believed to reflect the capture of an early stage of progression whereby individual resistant clones may be usefully ablated before significant systemic spread has occurred. The index report on this phenomenon employed an OPD definition of either any number of nonleptomeningeal CNS and/or 4 sites or fewer of extra-CNS progression on first-generation ALK or EGFR tyrosine kinase inhibitor (TKI) therapy. The report showed that 54% (15/28) of *ALK*+ and 43.5% (10/23) *EGFR*+ patients had oligo-progression on crizotinib or erlotinib that was deemed appropriate for local therapy (LT) and continuation of the same systemic drug treatment.^1^

LT while continuing on the same TKI resulted in a time to next progression (progression free survival 2, PFS2) of 6.2 months post-LT.^1^ Similar results were subsequently reported by other groups for LT to CNS^2^, ^3^, ^4^ and extra-CNS^3^^,^^5^ progression in patients with either *ALK*-rearranged or *EGFR* mutant NSCLC treated predominantly with first-generation drugs, with a median PFS2 ranging from 2.7 to 10 months.

In this study, we aimed to update our previous index report of LT for OPD with a newer, larger data set, more driver oncogenes (*EGFR, ALK, ROS1, BRAF, RET*), newer-generation TKIs with potentially greater CNS penetration, and longer follow-up. We also analyzed the pattern of disease progression in terms of CNS versus extra-CNS and number of sites after the first LT. Additionally, we assessed whether multiple clinical variables were associated with post-LT progression-free survival.

## Materials and Methods

### Study design and patient selection

Patients with histologically confirmed *EGFR/BRAF*-mutant (*EGFR+/BRAF+*) or *ALK*/*ROS1/RET-*rearranged (*ALK+/ROS1+/RET+*) metastatic NSCLC at the University of Colorado treated with at least 1 course of hypofractionated external-beam radiation therapy (≤15 fractions), stereotactic radiosurgery, or whole-brain radiation therapy while continuing to receive the same TKI between 2014 and 2020 were included. Data cutoff was August 10, 2021. As our clinical practice had changed since our prior study published in 2012, our practical definition of OPD potentially suitable for LT was either any number of nonleptomeningeal CNS sites and/or extra-CNS sites progressing on targeted therapy up to a total of 5 sites or fewer in total. Brain metastases were counted as one site regardless of number of individual brain metastases on both our prior study and this study. This was because of data supporting the use of SRS in up to 15 brain metastases and ongoing trials of up to 20 brain metastases.^6^ Additionally, in some cases, the number of brain metastases could not be quantified, and these were still amenable to WBRT while continuing with same TKI. The study was conducted with institutional review board approval (COMRIB #17-1004).

Baseline clinical characteristics were determined by retrospective electronic record review, including age at diagnosis, sex, smoking status, tumor histology, oncogenic driver, systemic therapy at the time of LT, date of start of systemic therapy, date of LT, number and location of sites treated with LT, dose and fraction of LT, date of systemic therapy change and number and location of sites of progression at time of local therapy change, date of last follow-up, and date of death.

Baseline and ongoing CNS and body imaging with magnetic resonance imaging (MRI), computed tomography (CT), and/or positron emission tomography (PET)/CT were performed according to physician discretion. In general, CT scans were performed every 3 months and MRIs of the brain were performed every 3 to 6 months depending on the presence or absence of known brain metastases. The decision to treat with LT or drug change was also at the discretion of the treating physician.

### Statistical analysis

Progression-free-survival 1 (PFS1) was calculated from time of initiation of targeted therapy to the first day of the first LT course on the targeted therapy. Subsequent PFS (eg, PFS2, PFS3) were calculated from the first day of each LT course to the first day of the next LT course on the same targeted therapy, change in systemic therapy, or death or loss to follow-up, whichever comes first. Extended-PFS was calculated from the first day of the first LT course to the day of change in systemic therapy, death, or loss to follow-up, whichever came first. Duration on treatment (DoT) was calculated from time of initiation of targeted therapy to time of switch of systemic therapy. For all endpoints, patients who were lost to follow-up or remained on the same TKI after LT by the end of the study were censored. Any radiation therapy that took place within 28 days before or after drug change without any radiographic evidence or documentation of clinical progression were mostly palliative in nature or preplanned together with decision of drug change and therefore were not counted as a course of LT for oligoprogression. As radiation therapy to different sites (eg, CNS and extra-CNS targets) were occasionally split into nonoverlapping courses, 2 courses of radiation therapy occurring within 28 days of each other without any radiographic evidence or documentation of clinical progression were considered concomitant and therefore counted as one single LT course with PFS time based on the first course of LT.

Outcomes were stratified based on age, sex, smoking history, stage at diagnosis, driver oncogenes, TKIs, line of systemic therapy, number of LT sites, and CNS versus extra-CNS sites for LT. Within each category, potential predictors were collapsed into limited subcategories due to sample size considerations. For TKIs, high and low CNS penetrance subcategories were used. TKIs with high CNS penetrance represented those reported to have a CNS objective response rate of ≥40%.^6^ In our study population, this included alectinib, brigatinib, ceritinib, entrectinib, lorlatinib, osimertinib, pralsetinib, and repotrectinib. Those with lower CNS penetrance included afatinib, crizotinib, dabrafenib/trametinib, erlotinib, gefitinib, mobocertinib, and rocilectinib. The number of lines of systemic therapies were collapsed into 2 subcategories: “first- or second-line” and “third-line or more.” The number of LT sites were subcategorized as “1 site” and “2 or more sites,” and stage at diagnosis was categorized as “stage I-III” and “stage IV.”

Kaplan-Meier (KM) curves were used to assess the median duration of PFS1, PFS2, and Extended-PFS. The corresponding 95% confidence intervals in the presence of censoring were also reported. Since the KM method does not account for the correlation between different TKIs within subjects, the KM analysis set was restricted to the first TKI per patient. For variables collected at the LT level, the observation corresponding to the end of the first LT was used to stratify the PFS2 and Extended-PFS definitions. For each primary time to event outcome, PFS was presented overall and then stratified separately for each predictor. Secondary PFS variables (PFS3, PFS4) were presented overall.

A multivariate frailty Cox model was used to model the association between PFS1/Extended-PFS and potential predictors. The frailty model allows multiple observations from the same subject by accounting for within-subject correlations. Predictors under consideration included oncogene, drug generation, number of lines of systemic therapy, CNS versus extra CNS at the first LT evaluation (Extended-PFS), and the number of LT sites at the first LT evaluation (Extended-PFS). The model adjusted for demographic variables to minimize the potential for confounding effects.

## Results

### Demographics

Demographics of the 89 eligible patients are summarized in Table 1. Median age at diagnosis was 61 years old. The majority of patients were female (65.2%) and never smoked (77.5%). All patients had adenocarcinoma subtype with the majority initially diagnosed as stage IV (87.6%). The majority of patients had *EGFR*+ (n = 55; 61.8%) or *ALK*+ (n = 25; 28.1%) NSCLC. There were 5 *ROS1*+, 3 *BRAF*+ and 1 *RET*+ cases. There were considered to be insufficient *BRAF*+ and *RET*+ cases to pursue *BRAF* or *RET* specific analyses. Each patient could receive LT multiple times on the same systemic therapy and while on a subsequent systemic therapy. In total, these patients received 184 courses of LT while on 113 lines of systemic therapies.

**Table 1:** Descriptive statistics of demographics and treatment details

		N (%)*
Age (years), median (range) (N = 89)		61 (22-85)
Age category (years)		
<65		60 (67.4%)
≥65		29 (32.6%)
Sex (N = 89)		
Male		31 (34.8%)
Female		58 (65.2%)
Smoking history (N = 89)		
Never smoked		69 (77.5%)
Former smoker		20 (22.5%)
Current smoker		0
NSCLC subtype (N = 89)		
Adenocarcinoma		89 (100.0%)
Stage at diagnosis (N = 89)		
I		2 (2.2%)
II		0
III		9 (10.1%)
IV		78 (87.6%)
Driver oncogenes (N = 89)		
EGFR		55 (61.8%)
Exon 19 deletion		25 (28.1%)
L858R		24 (27.0%)†
Exon 20 insertion		3 (3.4%)
G719X		3 (3.4%)‡
ALK		25 (28.1%)
ROS1		5 (5.6%)
BRAF V600E		3 (3.4%)
RET		1 (1.1%)
Tyrosine kinase inhibitors (N = 113)§		
EGFR TKI		66 (58.4%)
Afatinib		4 (3.5%)
Erlotinib		30 (26.5%)
Gefitinib		1 (0.9%)
Mobocertinib		3 (2.7%)
Osimertinib		24 (21.2%)
Rocilectinib		4 (3.5%)
ALK TKI		35 (31.0%)
Alectinib		5 (4.4%)
Brigatinib		13 (11.5%)
Ceritinib		1 (0.9%)
Crizotinib		13 (11.5%)
Lorlatinib		3 (2.7%)
ROS1 TKI		8 (7.1%)
Crizotinib		3 (2.7%)
Entrectinib		2 (1.8%)
Lorlatinib		2 (1.8%)
Repotrectinib		1 (0.9%)
BRAF V600E TKI		3 (2.7%)
Dabrafenib/trametinib		3 (2.7%)
RET		1 (0.9%)
Pralsetinib		1 (0.9%)
Line of systemic therapy (N = 113)^d^		
1		46 (40.7%)
2		39 (34.5%)
>2		28 (24.8%)
Number of LT course per systemic therapy line (N = 113)^d^		
1		64 (56.6%)
2		31 (27.4%)
3		14 (12.4%)
4		4 (3.5%)
Number of sites per LT course (N = 184)‖		
1		138 (75.4%)
2		32 (16.9%)
3		12 (6.6%)
4		1 (0.5%)
5		1 (0.5%)
Sites of LT (N = 246)		
Adrenal		4 (1.6%)
Bone		70 (28.5%)
Brain		70 (28.5%)
Chest wall		5 (2.0%)
Liver		9 (3.7%)
Lung		43 (17.5%)
Lymph node		38 (15.4%)
Pancreas		2 (0.8%)
Pericardium		1 (0.4%)
Pleura		4 (1.6%)

Site (n = 246)	Median dose in Gy (range)	Median number of fractions (range)
Adrenal (n = 4)	37.5 (35-45)	7.5 (3-10)
Bone (n = 70)	20 (8-40)	5 (1-10)
Brain (n = 70)	20 (18-37.5)	1 (1-15)
Chest wall (n = 5)	30 (24-40)	5 (3-10)
Liver (n = 9)	40 (24-50)	4 (3-10)
Lung (n = 43)	45 (20-54)	5 (3-20)¶
Lymph node (n = 38)	40 (24-50)	10 (3-15)
Pancreas (n = 2)	35 (30-40)	10 (10-10)
Pericardium (n = 1)	24 (24-24)	6 (6-6)
Pleura (n = 4)	42.5 (24-50)	5.5 (5-10)

Descriptive statistics are presented for 89 analyzable patients. Variables collected at the patient level have 89 observations, variables collected at the patient-drug level have 113 observations, and variables collected at the patient-drug-LT level have 184 observations. There are no missing observations.

⁎ Categorical variables are presented as the number and frequency (N(%)) in each category. All variables are categorical unless otherwise indicated.

† One patient had L858R and E709V. One patient had L858R and L747V.

‡ One patient had G719A and V834L.

§ This is different from number of patients because one patient can receive LT to more than one line of TKI

‖ Brain metastases are counted as one site regardless of number of individual brain metastases. Thoracic lymph nodes are also counted as one site regardless of number.

¶ One treatment was with 50 Gy over 20 fractions to the lung, which is outside our inclusion criteria (≤15 fractions). However, given it is local ablative intent, it was included in our data set.

### Treatment details

Treatment details are summarized in Table 1. Fifty-two lines (46.0%) of systemic therapy were considered to be with high-CNS-penetrant TKIs based on our definition. Eighty-five lines (75.2%) of systemic therapy were the first or second line of therapy. Most patients received only 1 course of LT on a given line of systemic therapy (56.6%). Typically, only 1 site was irradiated during a given course of LT (75.4%). Of the total 246 sites treated with LT, the most common sites were brain (28.5%) and bone (28.5%), followed by lung (17.5%) and lymph nodes (15.4%). The median and range of radiation dose and number of fractions are summarized in Table 1.

### Treatment outcomes

**Figure 1:**
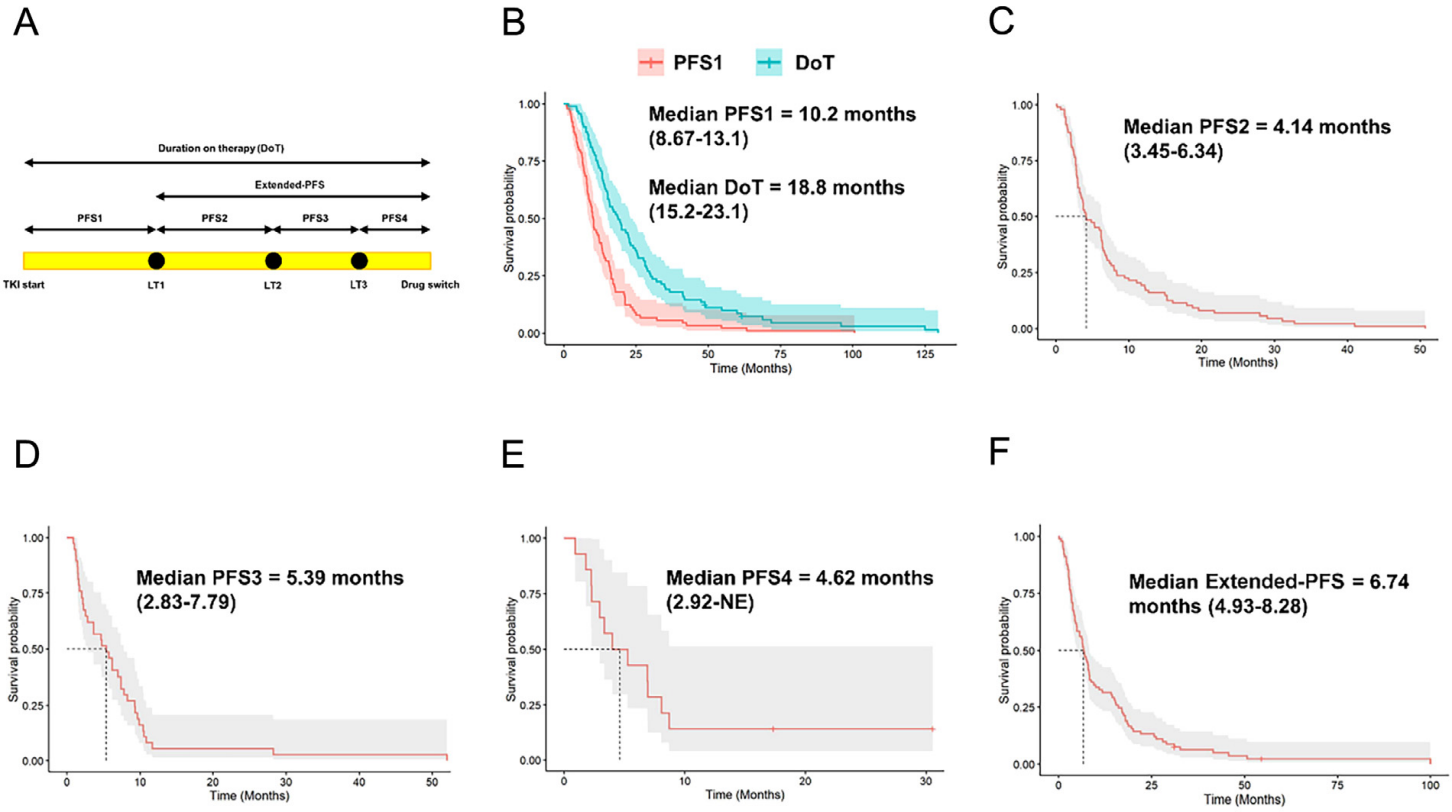
(A) Schematic diagram of definition of survival periods. (B-F) Kaplan-Meier curves for PFS1 and DoT (B), PFS2 (C), PFS3 (D), PFS4 (E), Extended-PFS (F).

Kaplan-Meier curves were used to assess the median PFS time on the first TKI according to each PFS definition. The ablations extended the median time on a TKI treatment by 6.7 months (95% CI, 4.9-8.3) according to the Extended-PFS definition. Median PFS1, measured from the time of TKI start to the first episode of LT, was 10.2 months (95% CI, 8.7-13.1). DoT, measured from time of TKI start to drug switch was 18.8 months (95% CI, 15.2-23.1). Median PFS2, measured from the time of first LT to subsequent LT or drug change, was 4.1 months (95% CI, 3.5-6.3). For patients who received second or third LT episodes, PFS3 and PFS4 were 5.4 months (95% CI, 2.8-7.8) and 4.6 (95% CI, 2.9-NE), respectively (Fig. 1).

### Patterns of progression and treatment after LT

**Figure 2:**
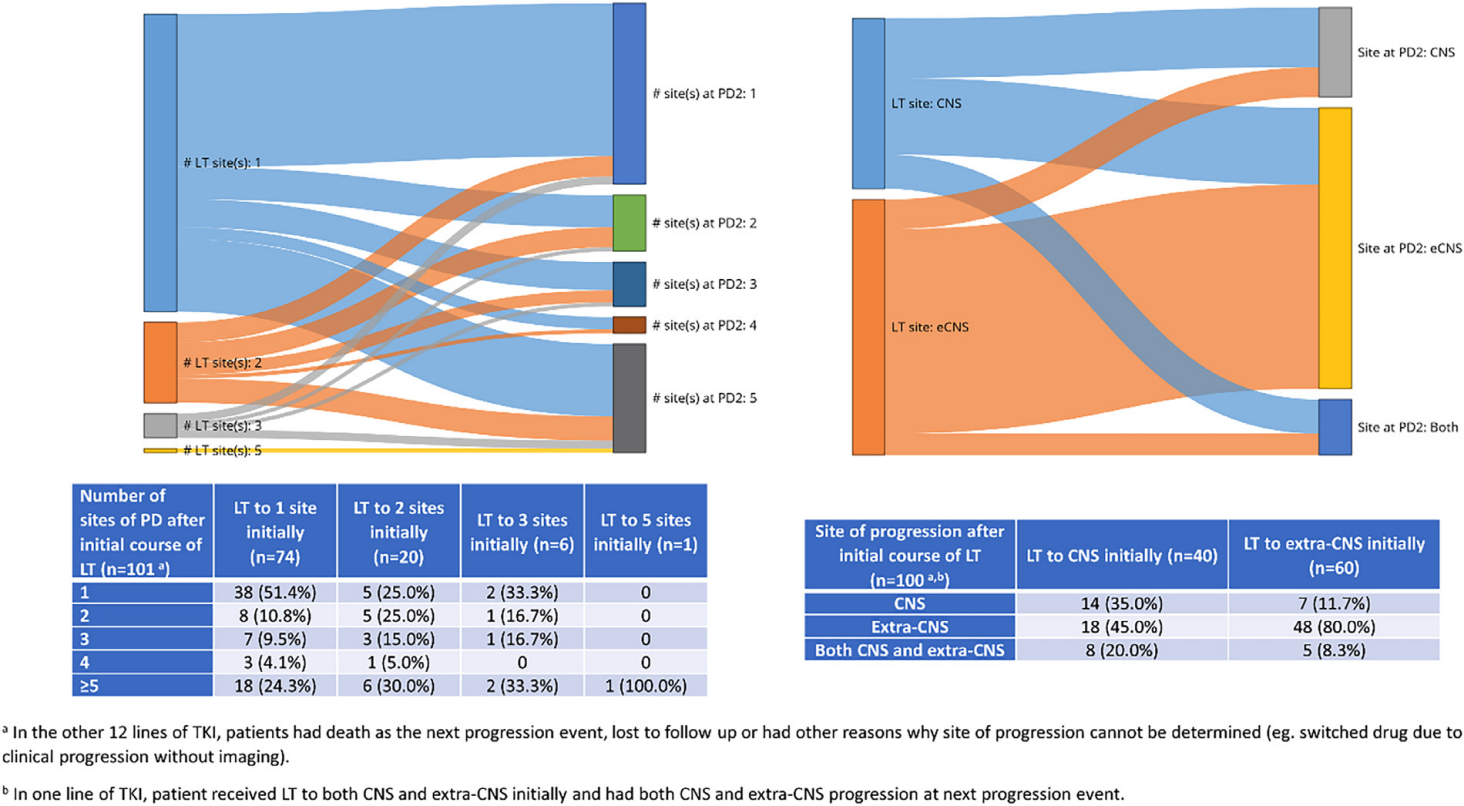
Sites of progression after first LT on a given systemic therapy.

We analyzed the pattern of progression after first LT (ie, at progression event 2). More than half of patients who underwent LT to a single site at the time of first progression had only 1 site on next disease progression (Fig. 2). Of the 40 patients who received LT to CNS at the time of first progression, 14 (35.0%) experienced CNS only progression at the next progression event, 18 (45.0%) had extra-CNS progression, and 8 (20.0%) experienced both (Fig. 2). For patients who had a drug change at the second progression event, 49.1% had ≥5 sites of disease progressing at that point, and 88.5% had extra-CNS disease progression (Table 2).

**Table 2:** Number and location of sites of disease progression for patients who had a change of drug at the next progression event after the first LT (n = 53)

Number of sites of progression	Frequency (%)
1	11 (20.8%)
2	7 (13.2%)
3	6 (11.3%)
4	2 (3.8%)
≥5	26 (49.1%)
Unknown*	1 (1.9%)
CNS vs extra-CNS progression at PD2	Frequency (%)
CNS	6 (11.3%)
Extra-CNS	36 (67.9%)
Both CNS and extra-CNS	10 (18.9%)
Unknown*	1 (1.9%)

⁎ Patient switched drug due to clinical progression without imaging.

### Factors affecting progression free survival

Multivariable frailty Cox models were used to examine variables (including age at diagnosis, sex, smoking history, stage at diagnosis, driver oncogene, high versus low CNS penetrant TKI, systemic therapy line, number of sites at first LT (Extended-PFS) and sites at LT 1 (Extended-PFS)) potentially associated with PFS1 or Extended-PFS to allow 2 or more lines of systemic therapy from a subject by accounting for within-subject correlation. In this model, all 113 lines of systemic therapy during which LT was performed were included. However, for strata with 3 or fewer observations as well as rechallenges with the same TKI for the same patients, data for the entire line of systemic therapy were excluded. This resulted in a total sample size of 104 lines of systemic therapy. Multivariable analyses did not reveal any statistically significant association between PFS1 or Extended-PFS and any predictor (Table 3).

**Table 3:** Multivariate associations according to the Frailty Cox model

PFS1		
Main effects	HR (95% CI)	*P* value
**Age (years)**	0.99 (0.97-1.01)	.36
**Sex**		
Male vs female	1.65 (0.90-3.03)	.11
**Smoking history**		
Never smoked vs smokers (former/current)	0.87 (0.43-1.75)	.69
**Stage at diagnosis**		
IV vs I-III	1.09 (0.46-2.59)	.84
**Driver oncogenes**		
EGFR vs ROS1	2.01 (0.64-6.28)	.23
ALK vs ROS1	1.33 (0.41-4.34)	.64
**Tyrosine kinase inhibitors**		
High-CNS-penetrant vs low-CNS-penetrant	0.78 (0.45-1.34)	.36
**Systemic therapy line**		
>2L vs 1/2L	1.37 (0.66-2.86)	.40

Extended-PFS		
Main effects	HR (95% CI)	*P* value
**Age (years)**	0.99 (0.97-1.02)	.64
**Sex**		
Male vs Female	1.04 (0.52-2.05)	.92
**Smoking history**		
Never smoked vs smokers (former/current)	0.71 (0.33-1.54)	.39
**Stage at diagnosis**		
IV vs I-III	1.78 (0.64-4.98)	.27
**Driver oncogenes**		
EGFR vs ROS1	0.88 (0.23-3.33)	.85
ALK vs ROS1	0.92 (0.23-3.66)	.91
**Tyrosine kinase inhibitors**		
High-CNS-penetrant vs low-CNS-penetrant	0.59 (0.32-1.08)	.09
**Systemic therapy line**		
>2L vs 1/2L	0.88 (0.38-2.02)	.76
**Number of sites at LT #1**		
>1 vs 1	1.33 (0.63-2.77)	.45
**Sites at LT #1**		
extra-CNS vs CNS	1.66 (0.82-3.34)	.16

**Figure 3:**
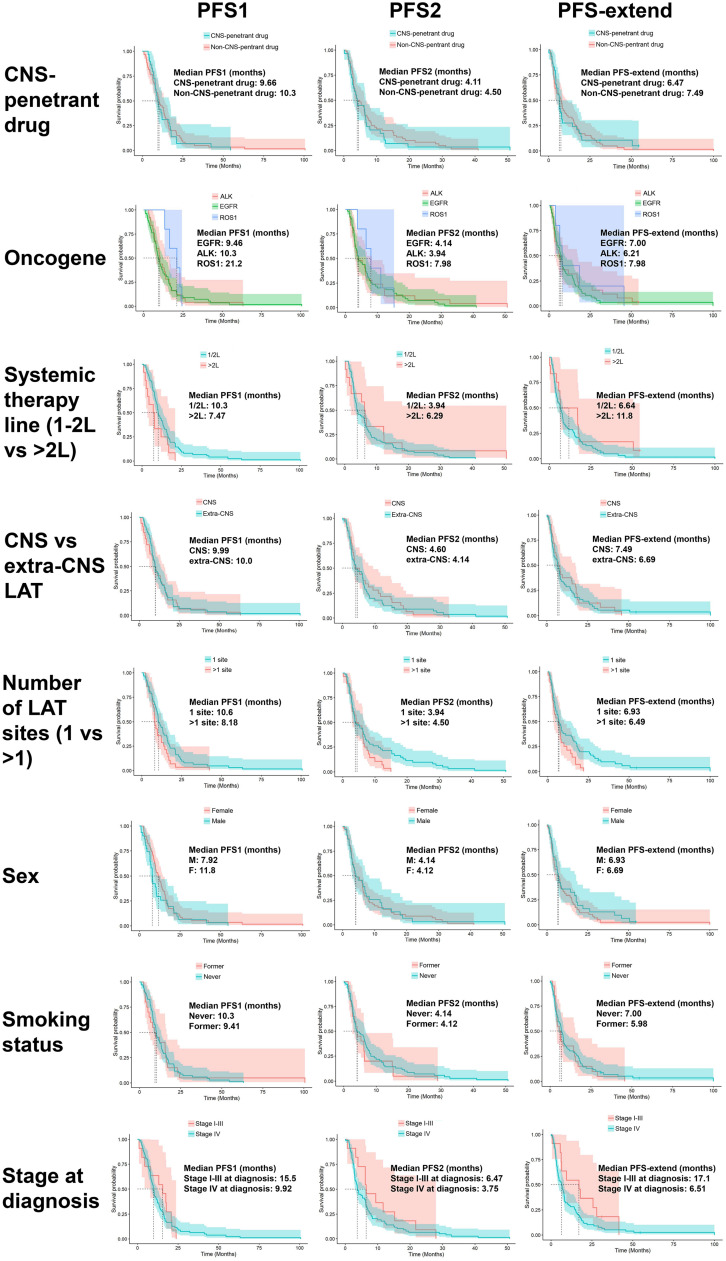
Kaplan-Meier curves of each time to event outcome, stratified by (A) demographic and clinical factor and (B) LT related factors.

We also estimated the median PFS intervals for each of these strata for the first TKI of each patient using K-M estimate (Fig. 3). For Extended-PFS, at 12 months the progression-free survival probability was 36.1% (95% CI, 25.8%-50.4%) for patients with 1 LT site at first progression and 21.4% (95% CI, 10.5%-43.6%) for patients with more than 1 LT site at first progression, but this did not reach statistical significance in the Cox frailty model (HR, 1.33 [0.63-2.77], *P* = .45). The Extended-PFS probability at 24 months for patients with 1 LT site at first progression was 19.7% (95% CI, 11.8%-32.7%), whereas no patients with more than 1 LT site at first progression remained at risk at 24 months.

## Discussion

The efficacy of LT for OPD in patients with *EGFR*+ and *ALK*+ NSCLC was first reported in 2012.^1^ Since then, several studies have shown similar efficacy using this approach, particularly for those with *EGFR*+ NSCLC and for first-generation TKIs.^3^, ^4^, ^5^^,^^7^, ^8^, ^9^, ^10^, ^11^ Partly because of challenges in determining an appropriate control arm, while a number of randomized controlled trials (RCT) have demonstrated the efficacy of upfront radiation therapy of oligo-metastatic disease^12^^,^^13^ and consolidative radiation therapy of oligoresidual disease on treatment,^14^^,^^15^ currently no large RCT data are available on the efficacy of LT for OPD. One relatively small study showed that local radiation therapy while continuing EGFR-TKI resulted in a significantly longer PFS2 (median PFS 7.0 months vs 4.1 months, *P* = .0017) and OS (OS 28.2 months vs 14.7 months, *P* = .026), compared with a matched cohort that was switched to chemotherapy.^8^ In addition, a study by Le et al showed that PFS2 for patients with *EGFR*-mutant NSCLC who received radiation therapy at progression was longer than those who did not receive radiation but continued to receive osimertinib after first progression (15.5 vs 8.2 months, *P* = .05).^16^

Our data, predominantly involving modern next-generation TKIs, continue to support the benefit of LT for OPD in oncogene-addicted NSCLC. There are very little data for LT for OPD in other oncogene-driven NSCLC. Our study adds to the current body of literature and includes updates on newer-generation TKIs and other oncogenes. Although the numbers for *ROS1, BRAF,* and *RET* were modest in our study, the data suggest the possibility of similar PFS extensions with LT across different oncogenic drivers (median Extended-PFS for EGFR = 7.00 months, ALK = 6.21 months and ROS1 = 7.98 months).

Using a similar analysis of a surrogate endpoint for radiographic progression, a recent study reported in a cohort of patients with stage IV *EGFR*+ NSCLC having ≤5 sites of OPD while on EGFR-TKI, the time between LT to further progression that led to stop of EGFR-TKIs was 6.9 months.^17^ This was very similar to our results of a median Extended-PFS of 6.74 months. In addition, our results also showed that multiple courses of LT can be performed on the same systemic therapy, with similar PFS extension on repeated treatments, and this strategy could be repeated over multiple lines of systemic therapies.

In our cohort, isolated CNS progression was more common after LT to CNS than after LT to extra-CNS sites on first progression (35.0% vs 11.7%; Table 2). Although it is expected that tumors that have demonstrated the ability to spread to the CNS would be more likely to manifest subsequent CNS progression, the degree to which potential detection biases in the setting of more frequent MRI imaging contributes to the observed differences is unknown. Compared with extra-CNS progression, isolated CNS progression in the setting of oncogene-addicted NSCLC might be due to inadequate drug exposure as opposed to clonal evolution of the tumor.^18^ Our subgroup analysis did not reveal any statistical difference in PFS2 or Extended-PFS whether a CNS site or an extra-CNS site was the first site of disease progression requiring LT.

Data from our cohort also suggested that a smaller number of progressing sites requiring LT was associated with fewer sites at the next progression event, although this should be interpreted with caution due to the small number of progression events with more than 2 lesions treated with LT in our cohort. About half of those who had a single site of disease at the first progression event had a single site of progression at the next event. Notably, 19.7% of patients with a single site ablated on first LT were able to remain on the same TKI at 24 months, while none of those in the group with more than 1 LT site did. Other studies also demonstrated better outcomes for patients who had LT to a single site versus multiple sites. In one study by Xu et al, patients with advanced *EGFR*+ NSCLC treated with TKI and a single metastatic site at baseline had a significantly longer time to first progression (median, 11.7 months vs 9.9 months, *P* < .001) and longer duration on TKI (median, 19.8 months vs 16.7 months, *P* = .001), compared to those with more than 1 metastasis.^3^ Another study also found a nonstatistically significant trend that the time from LT to next progression is longer for those who received LT to 1 to 2 sites compared with 3 to 4 sites of extra-CNS OPD at first progression (7 mo vs 2 mo, *P* = .12).^19^

There are several limitations to our study. First, this is a single-arm retrospective study in a single institution and therefore may be subject to selection bias. Second, we used the surrogate endpoints of time of LT and drug change instead of disease progression by RECIST criteria, which would be difficult to assess objectively in a retrospective study. Third, although it is standard to perform a CT scan of the body every 3 months, the exact timing was at the discretion of the treating physician. Moreover, the frequency of brain MRI used may have depended on the patient's prior history of brain metastases, therefore potentially contributing some detection bias for subsequent brain metastases. Fourth, the decision between LT and drug change was at the discretion of the treating physician. As shown in Table 2, only 49.1% of patients who underwent drug change at the second progression event had ≥5 sites of disease progression, and 20.8% of patients had a single site of disease progression. The reasons these patients underwent drug change instead of LT may have varied, but 2 of the most common reasons noted included the identification of a well-tolerated effective next-line systemic option (such as identification of an *EGFR* T790M mutation allowing for switch to osimertinib), and that the site of progression was not amenable to radiation (such as a pleural effusion). Fifth, although our study focused on the role of radiation therapy as the modality of LT, we also acknowledge there are other modalities of local therapy such as surgery and radio frequency ablation. Sixth, our definition of LT is based on the parameter of radiation therapy ≤15 fractions while continuing to receive the same TKI. It is therefore possible that some of the treatment captured included could have been of palliative intent given that the intent of the treatment was not discernible from our database.

Another caveat is that patients who manifest OPD on therapy suitable for LT may have more favorable tumor biology. Although our first publication showed that time of first progression on drug was comparable between those who were eligible for LT and those who were not (9.8 months vs 12.8 months),^1^ a subsequent study focused on *ALK*+ patients showed those who were eligible for LT had a median PFS1 of 14 months, compared with 7.2 months for those who were not eligible for LT.^19^ Another study also found that patients in the local therapy group had a nonstatistically significantly longer time to progression on EGFR TKI therapy before local therapy, compared with patients who went on to receive systemic therapies only (*P* = .09).^5^

In contrast to oncogene-addicted NSCLC where OPD might be driven by emergence of defined on-target or off-target resistance mechanisms, OPD in patients treated with immune checkpoint inhibitors or chemotherapy might be driven by different mechanisms such as immune tolerance or changes in tumor environment, and, as such, the underlying biologic rationale for LT in the context of oncogene-addicted NSCLC (eg, as a means of eliminating an evolutionary reservoir of resistant subclones)^20^ may or may not directly translate to NSCLC without oncogenic drivers. However, a recent study showed that in patients with NSCLC of which the majority (86%) had no oncogenic driver who had OPD of ≤5 sites, the addition of SBRT to standard of care systemic therapy resulted in an improvement in PFS compared with standard of care systemic therapy alone (10 months vs 2.2 months; *P* = .002).^21^ This suggests the strategy of LT for OPD on other therapies may be similarly applicable to patients with no oncogenic driver.

In summary, our results further support the efficacy of LT for OPD across oncogene-addicted NSCLC in the era of newer generations of TKIs and multiple different molecular drivers. We showed that LT can be repeatedly used to extend the duration of any given line of systemic therapy and may be particularly beneficial for patients with a single site of OPD. However, a prospective study standardizing the criteria for LT for OPD versus a relevant drug change across different molecular subtypes of NSCLC, with standard frequency and modality of imaging, across multiple centers would be required to delineate the true extent of benefit for this treatment approach. Ongoing randomized studies such as the STOP trial (NCT02756793), PROMISE-004 (NCT03808662), and HALT (NCT03256981) may shed light on some of these questions.
